# Delta-8-THC: Delta-9-THC’s nicer younger sibling?

**DOI:** 10.1186/s42238-021-00115-8

**Published:** 2022-01-04

**Authors:** Jessica S. Kruger, Daniel J. Kruger

**Affiliations:** 1 Department of Community Health and Health Behavior, University at Buffalo, SUNY, 319 Kimball Tower, Buffalo, NY USA; 2 Population Studies Center, Institute for Social Research, University of Michigan, 426 Thompson St, Ann Arbor, MI USA

**Keywords:** Medical cannabis, Cannabis, Cannabinoid, Delta-8-THC, Subjective effects

## Abstract

**Background:**

Products containing delta-8-THC became widely available in most of the USA following the 2018 Farm Bill and by late 2020 were core products of hemp processing companies, especially where delta-9-THC use remained illegal or required medical authorization. Research on experiences with delta-8-THC is scarce, some state governments have prohibited it because of this lack of knowledge.

**Objective:**

We conducted an exploratory study addressing a broad range of issues regarding delta-8-THC to inform policy discussions and provide directions for future systematic research.

**Methods:**

We developed an online survey for delta-8-THC consumers, including qualities of delta-8-THC experiences, comparisons with delta-9-THC, and open-ended feedback. The survey included quantitative and qualitative aspects to provide a rich description and content for future hypothesis testing. Invitations to participate were distributed by a manufacturer of delta-8-THC products via social media accounts, email contact list, and the Delta8 Reddit.com discussion board. Participants (*N* = 521) mostly identified as White/European American (90%) and male (57%). Pairwise *t* tests compared delta-8-THC effect rating items; one-sample *t* tests examined responses to delta-9-THC comparison items.

**Results:**

Most delta-8-THC users experienced a lot or a great deal of relaxation (71%); euphoria (68%) and pain relief (55%); a moderate amount or a lot of cognitive distortions such as difficulty concentrating (81%), difficulties with short-term memory (80%), and alerted sense of time (74%); and did not experience anxiety (74%) or paranoia (83%). Participants generally compared delta-8-THC favorably with both delta-9-THC and pharmaceutical drugs, with most participants reporting substitution for delta-9-THC (57%) and pharmaceutical drugs (59%). Participant concerns regarding delta-8-THC were generally focused on continued legal access.

**Conclusions:**

Delta-8-THC may provide much of the experiential benefits of delta-9-THC with lesser adverse effects. Future systematic research is needed to confirm participant reports, although these studies are hindered by the legal statuses of both delta-8-THC and delta-9-THC. Cross-sector collaborations among academics, government officials, and representatives from the cannabis industry may accelerate the generation of knowledge regarding delta-8-THC and other cannabinoids. A strength of this study is that it is the first large survey of delta-8 users, limitations include self-report data from a self-selected convenience sample.

**Supplementary Information:**

The online version contains supplementary material available at 10.1186/s42238-021-00115-8.

## Background

Among hundreds of cannabinoids, delta-8-tetrahydrocannabinol (delta-8-THC, Δ^8^-THC) has rapidly risen in popularity among consumers of cannabis products. Delta-8-THC is an isomer or a chemical analog of delta-9-THC, the molecule that produces the experience of being high when ingesting cannabis (Qamar et al. 2021). Delta-8-THC differs in the molecular structure from delta-9-THC in the location of a double bond between carbon atoms 8 and 9 rather than carbon atoms 9 and 10 (Razdan 1984). Due to its altered structure, delta-8-THC has a lower affinity for the CB1 receptor and therefore has a lower psychotropic potency than delta-9-THC (Hollister and Gillespie 1973; Razdan 1984). Delta-8-THC is found naturally in Cannabis, though at substantially lower concentrations than delta-9-THC (Hively et al. 1966). It can also be synthesized from other cannabinoids (e.g., Hanuš and Krejčí 1975).

The 2018 Farm Bill did not specifically address delta-8-THC, but effectively legalized the sale of hemp-derived delta-8-THC products with no oversight. Its popularity grew dramatically in late 2020, gaining the attention of cannabis consumers and processors throughout the United States. As of early 2021, delta-8-THC is considered one of the fastest-growing segments of hemp derived products, with most states having access (Richtel 2021). Yet, little is known about experiences with delta-8-THC or effects in medical or recreational users (Hollister and Gillespie 1973; Razdan 1984).

In 1973, delta-8-THC and delta-9-THC were administrated to six research participants. Despite the small sample size, researchers concluded that delta-8-THC was about two-thirds as potent as delta-9-THC and was qualitatively similar in experiential effects (Hollister and Gillespie 1973; Razdan 1984). In 1995, researchers gave delta-8-THC to eight pediatric cancer patients two hours before each chemotherapy session. Over the course of 8 months, none of these patients vomited following their cancer treatment. The researchers concluded that delta-8-THC was a more stable compound than the more well-studied delta-9-THC (Abrahamov et al. 1995, consistent with other findings (Zias et al. 1993), and suggested that delta-8-THC could be a better candidate than delta-9-THC for new therapeutics.

In recent months, 14 U.S. States have blocked the sale of delta-8-THC due to the lack of research into the compound’s psychoactive effects (Sullivan 2021). All policies and practices, including those related to substance use and public health, should be informed by empirical evidence. The current study seeks to better understand the experiences of people who use delta-8-THC to inform policy discussions and provide directions for future systematic research. Because this is the first large survey of delta-8-THC consumers, we take an exploratory approach to describe experiences with delta-8-THC. We combine quantitative rating items with open-ended qualitative items enabling participants to provide feedback which is rich in content.

## Methods

### Procedures

We developed an anonymous Qualtrics online survey to assess experiences with delta-8-THC. Bison Botanics, a manufacturer of delta-8-THC and CBD products in New York State, distributed invitations to participate in the study via their social media accounts (Facebook, Instagram), via their email contact list, and via the Delta8 online discussion board (Subreddit) on Reddit.com. The invitation read, “Are you a Delta-8-THC consumer? We’ve partnered with researchers at the University at Buffalo and the University of Michigan to learn more about experiences with delta-8-THC and its impact on public health and safety.” Screening questions verified that participants were 18 years of age or older, were currently in the USA, and used or consumed products containing delta-8-THC. Surveys were completed between June 12 and August 2, 2021. Delta-8-THC products were sold legally in New York State until July 19, 2021.

### Participants

Completed surveys (*N* = 521) were included for analyses, the completion rate was 74%. Participants were men (57%), women (41%), and individuals who reported another gender identity (2%). The mean age was 34 years old (SD = 11, range: 18–76). Participants had completed 15 years of education on average (SD = 2, range: 8–20), 17% were currently students. Participants identified (inclusively) as White/European American (90%), Hispanic/Latino (5%), Black/African American (3%), American Indian or Alaska Native (3%), Asian (3%), Native Hawaiian/Pacific Islander (1%), and Other (3%). Most (59%) participants provided ZIP Codes, which ranged across 38 U.S. States. The largest portion was from New York State (29%), all other states were below 10%. Nearly all these participants (90%) were in states where delta-9-THC Cannabis products were not yet commercially available for adult (i.e., “recreational”) use.

### Measures

Participants reported on the content of their experiences with delta-8-THC by rating its effects. The question stem read: “Please indicate how much you experience the following when you use delta-8-THC:” Specific aspects were altered sense of time; anxiety (unpleasant feelings, nervousness, worry); difficulty concentrating; difficulties with short-term memory; euphoria (pleasure, excitement, happiness); pain relief; paranoia (thinking that other people are out to get you, etc.); and relaxation. Response options were not at all, a little, a moderate amount, a lot, a and great deal.

Two items assessed participants’ comparisons of experiences with delta-8-THC and delta-9-THC. The first question read: “How does Delta-8-THC compare to Delta-9-THC in the *intensity or strength* of effects?” [emphasis in original]; with response options: Delta-8-THC is much more intense, Delta-8-THC is somewhat more intense, about the same, Delta-9-THC is somewhat more intense, Delta-9-THC is much more intense, do not know. The second question read: “How does Delta-8-THC compare to Delta-9-THC in the *duration or length* of effects?” [emphasis in original]; with response options: Delta-8-THC lasts a lot longer, Delta-8-THC lasts a little longer, about the same, Delta-9-THC lasts a little longer, Delta-9-THC lasts a lot longer, do not know.

Participants were asked the open-ended question, “Do you have any comments about how Delta-8-THC compares to Delta-9-THC?” after the rating items. This item was followed by a brief demographic section assessing age, gender identity, education, ethnicity, and ZIP Code. At the end of the survey participants were asked: “Do you have any comments about these topics or this survey?” There were no restrictions on participants’ responses.

### Analysis

**Fig. 1:**
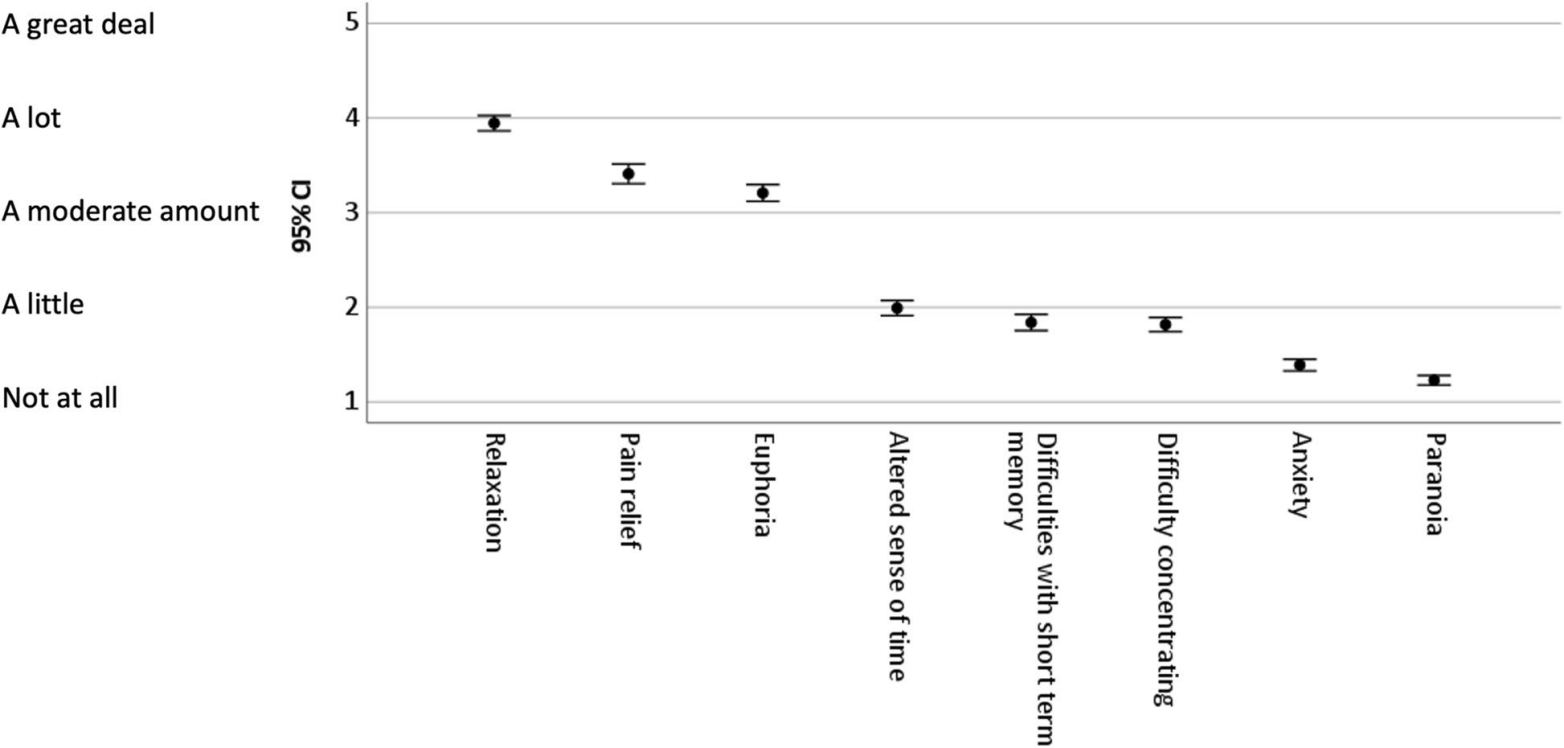
Participant experiences with delta-8-THC. *Note*: values are means with 95% confidence intervals. Experiences were coded as: 1 = not at all, 2 = a little, 3 = a moderate amount, 4 = a lot, 5 = a great deal

**Fig. 2:**
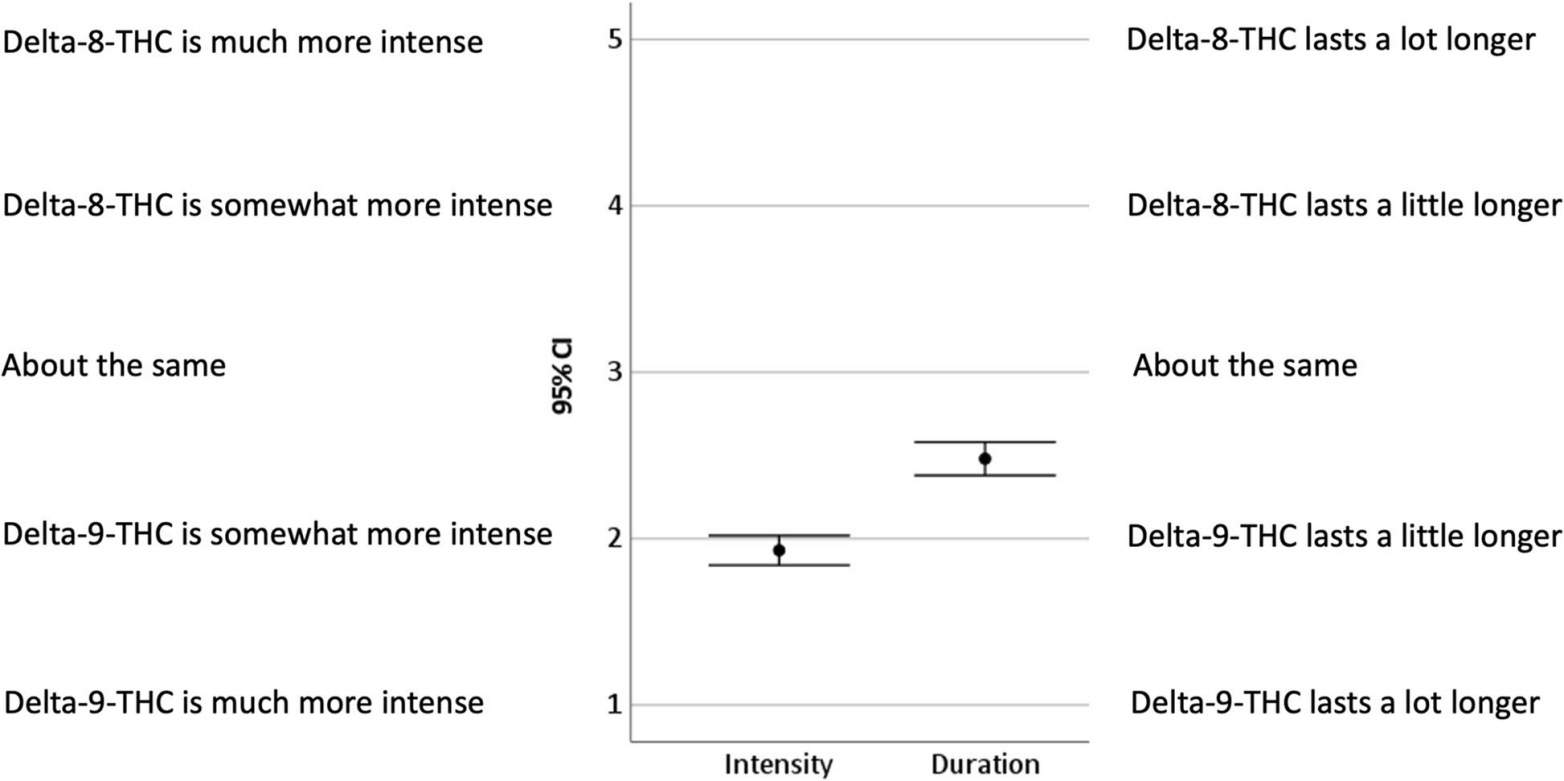
Participant comparisons of delta-8-THC and delta-9-THC experiences. *Note*: values are means with 95% confidence intervals. Intensity was coded as: 1 = Delta-9-THC is much more intense, 2 = Delta-9-THC is somewhat more intense, 3 = about the same, 4 = Delta-8-THC is somewhat more intense, 5 = Delta-8-THC is much more intense. Duration was coded as: 1 = Delta-9-THC lasts a lot longer, 2 = Delta-9-THC lasts a little longer, 3 = about the same, 4 = Delta-8-THC lasts a little longer, 5 = Delta-8-THC lasts a lot longer

Pairwise *t* tests compared ratings on delta-8-THC effect items; descriptive statistics, 95% confidence intervals, and effect sizes were calculated (see Table 1 and Fig. 1). Responses to items comparing delta-8-THC to delta-8-THC intensity and duration were examined by one-sample *t* tests with a comparison value of 3 (“About the same”), effect sizes and 95% Confidence Intervals were calculated (see Fig. 2). Demographic comparisons were made for participants’ gender with between-subjects *t* tests, participants’ age with Pearson correlations, and participants’ educational levels with partial correlations controlling for age. Responses to open-ended questions were coded as a set to avoid the duplication of codes for the same participant (see Table 2). The coders have been trained in qualitative methods and an inductive coding method was used to create a codebook. After the first coder assigned the codes, a line-by-line coding was used to then categorize codes. To establish interrater reliability, two coders independently read participant responses and identified overall themes. Once general themes were established, the responses were coded for theme categories and subcategories. Coding discrepancies were resolved, coding omissions were eliminated by adding codes, although no previously identified themes were deleted. Instances of themes and subthemes were calculated across participants. Individual participants could express more than one subtheme within a thematic category.

**Table 1:** Comparison of effects from Delta-8 THC with item descriptive statistics

Effect	*M*	SD	2	3	4	5	6	7	8
1. Relaxation	3.96	0.92	0.45	0.74	1.58	1.60	1.72	2.06	2.26
2. Pain relief	3.41	1.17	–	0.16	1.04	1.06	1.12	1.44	1.68
3. Euphoria	3.22	0.99		–	1.08	1.16	1.17	1.49	1.81
4. Altered sense of time	2.00	0.91			–	0.15	0.18	0.62	0.82
5. Difficulties with short-term memory	1.84	0.95				–	0.01°	0.44	0.62
6. Difficulty concentrating	1.83	0.85					–	0.49	0.67
7. Anxiety	1.38	0.70						–	.027
8. Paranoia	1.22	0.56							–

*Note*: Values in columns 2–8 indicate effect sizes for pairwise comparisons, *d* = .20 indicates a small effect, *d* = .50 indicates a medium effect, *d* = .80 indicates a large effect. ° indicates *p* = .405, all other comparisons are *p* < .001

**Table 2:** Themes in responses to open-ended questions

Theme/subtheme	Count
Comparisons between Delta-8 THC and Delta-9 THC	239
Overall similarity of experience	38
Delta-8 THC is less intense or potent than Delta-9 THC	38
Delta-8 THC enables better mental clarity than Delta-9 THC	37
Delta-8 THC produces less anxiety than Delta-9 THC	28
Delta-8 THC produces less paranoia than Delta-9 THC	20
Delta-8 THC has a lower cost than Delta-9 THC	11
Delta-8 THC is more accessible than Delta-9 THC	7
Prefers Delta-8 THC	6
Delta-8 THC has a shorter duration of effect than Delta-9 THC	6
Delta-8 THC produces less sedation than Delta-9 THC	5
Can be more active and/or productive with Delta-8 THC	5
Prefers Delta-9 THC	4
Delta-8 THC is legal so no risk of arrest, job loss, etc.	3
Delta-8 THC is harsher on the lungs when inhaled	3
Delta-8 THC has better social acceptance	3
Delta-8 THC provides better pain relief	3
Delta-8 THC stimulated the appetite less than Delta-9 THC	3
Delta-8 THC generates fewer or no panic attacks	2
Delta-8 THC provides better relaxation	2
Delta-8 THC has lesser (unspecified) adverse effects	2
Therapeutic effect or benefit from Delta-8 THC	62
Relaxation	15
Pain relief	9
Anxiety	9
Sleep aid	6
Maintaining a positive mood	4
Post-traumatic stress disorder (PTSD)	3
Depression	3
Migraines	2
Increasing appetite	2
Comments on the study or researchers	33
Praise for the researchers for conducting a study on Delta-8	22
Feedback and suggestions on the survey content or features	9
Criticism of study design	2
Expressions of concern	22
Concern for continued legal access to Delta-8 THC	17
Concern for the purity of the product	3
General expressions of praise for Delta-8 THC	19
Substitution of Delta-8 THC for other substances	17
Cannabis and cannabis products containing Delta-9 THC	6
Comparisons between Delta-8 THC and pharmaceutical drugs	11
Delta-8 THC is better at pain relief	3
Dual use of Delta-8 THC and Delta-9 THC	8
Delta-8 THC is for working and being active and Delta-9 THC is for fun, relaxation, and recreation	5
Adverse effects of Delta-8 THC	6
Anxiety (at high doses)	2
Headache	2
Other comments	25
Delta-8 THC Edibles or tinctures are more potent than vaping	7
Building tolerance to Delta-8 THC	3
Biochemistry of Delta-8 THC	2
Delta-8 THC is more for medical use than recreational use	2
Recreational use of Delta-8 THC	2
Delta-8 THC is better than alcohol	2
Does not like Delta-8 THC	2

See Supplementary Table S1 for unique responses

## Results

Participants mostly consumed delta-8-THC through edibles (64%; brownies, gummies, etc.), vaped concentrates (48%; hash, wax, dabs, oil, etc.), and tinctures (32%). Some participants consumed delta-8-THC through smoking concentrates (23%; hash, wax, dabs, oil, etc.), smoking bud or flower (18%), vaping bud or flower (9%), topical products (9%; lotion, cream, oil, patch on skin), capsules (6%), suppositories (1%), and other methods (1%). Most participants (83%) also reported consuming delta-9-THC cannabis and products and reported substitution for delta-9-THC (57%) and pharmaceutical drugs (59%).

Experiences with delta-8-THC were most prominently characterized by relaxation, pain relief, and euphoria (see Table 1 and Fig. 1). Participants reported modest levels of cognitive distortions such as an altered sense of time, difficulties with short-term memory, and difficulty concentrating. Participants reported low levels of distressing mental states (anxiety and paranoia). There were large statistical effect sizes in differences between items in the first set of experiences (relaxation, pain relief, and euphoria) and items in the second set (cognitive distortions), and medium statistical effect sizes in differences between cognitive distortions and anxiety and paranoia.

On average participants reported that the effects of delta-8-THC were less intense, *t*(433) = 23.86, *p* < .001, *d* = 1.15, and had a shorter duration, *t*(421) = 10.08, *p* < .001, *d* = 0.49, than the effects of delta-9-THC (see Fig. 2). Proportionally, participants reported the intensity of effect as much more with delta-9-THC (36%), somewhat more with delta-9-THC (44%), about the same (15%), somewhat more with delta-8-THC (4%), and much more with delta-8-THC (2%). Proportionally, participants reported the duration of effect as much more with delta-9-THC (20%), somewhat more with delta-9-THC (27%), about the same (41%), somewhat more with delta-8-THC (8%), and much more with delta-8-THC (5%).

Demographic analyses indicated that women perceived delta-8-THC effects to be somewhat more intense, *t*(420) = 3.55, *p* < .001, *d* = 0.36, and longer lasting, *t*(408) = 3.45, *p* < .001, *d* = 0.36, compared to delta-9-THC than did men. Older individuals perceived delta-8-THC effects to be somewhat more intense, *r*(429) = .141, *p* = .003, and longer lasting, *r*(418) = .293 *p* < .001, compared to delta-9-THC than younger individuals. Controlling for age, those completing more years of education perceived delta-8-THC effects to be somewhat more intense, *r*(383) = .158, *p* = .003, and longer lasting, *r*(383) = .139 *p* = .006, compared to delta-9-THC than those with less education.

Participants (*n* = 204) provided text responses in one or both open-ended questions (see Table 2 and S1). The most common theme was comparisons between delta-8-THC and delta-9-THC. Participants’ responses containing this theme included: “Delta 8 feels like Delta 9’s nicer younger sibling”; “It has all the positives and many fewer drawbacks/side effects. It is less impairing and much less likely to cause anxiety or paranoia. It has much milder to nonexistent aftereffects”; “Delta 8 is not as heavy as Delta 9. With Delta 8, I am able to perform my normal day to day activities, i.e., no couch lock, paranoia, munchies. I am able to function well at work under the influence of Delta 8 whereas under the influence on Delta 9 at work, I am paranoid and feel less motivated to do work activities. Delta 8 has more of just a euphoria feeling than any other feeling for me. I want to do activities and I want to have a pleasurable time. Whereas if I have too much of Delta 9, all I want to do is watch TV, eat snacks, distance myself from the outside world. Delta 9 is better for sleep.”

The second most common theme was the therapeutic effect or benefit from delta-8-THC, participants’ responses containing this theme included: “It is like “lite” Delta 9. I can focus and work more with Delta 8 than Delta 9. It helps my pains and relaxation and I feel more able. Depending on the strain it has taken the place of melatonin for sleep.”; “As with any newer drug with limited study, care should be taken with its use. But I’ve personally found it immensely useful and therapeutic, with management of anxiety and sleep issues. Which nothing but far more addictive drugs (regarding anxiolytics), have helped with in the past. I hope lots more studies will be able to be done.”; “Delta-9 I pretty regularly experience panic attacks. Delta-8 I do not and it relieves symptoms of PTSD and anxiety pretty quickly.” The third most common theme was comments on the study or researchers. Some examples of this praise are “I'm glad that there's more academic research being done on the subject, thank you for doing it!” and “Keep up the good work. Need more studies and information on cannabinoids.”

The fourth most common theme was expressions of concern, particularly for continued legal access to delta-8-THC. Participants’ responses containing this theme included: “D8 is Great for daytime relief when you need to get stuff done. It has helped me a lot! I HOPE THEY DON’T BAN IT!”; “I feel that delta-8-THC is a very effective alternative to delta-9-THC with less side effects. I primarily consume it in combination with high CBD or CBG hemp. I do wish there was regulation purely for safety concerns; more reliable lab testing, testing specifically for solvents and reagents used in delta-8-THC production, etc. But I do fear that harsh regulation may get in the way of a wonderful substance that could improve the lives of many people. I hope against hope that a fear mongering campaign doesn't put an end to the golden age of D8 that we are currently experiencing.”

The fifth most common theme was general expressions of praise for delta-8-THC. Many participants had similar statements such as “Delta 8 is a great thing. It needs to stay accessible and affordable for the people that can really benefit.” The sixth most common theme was substitution of delta-8-THC for other substances. One participant stated: “The therapeutic and medicinal effects of Delta 8 have significantly improved my life, treating pain and sleeplessness while not making me feel the high I get from Delta 9. I have stopped taking pharmaceutical drugs and my health and wellbeing has improved.”

The seventh most common theme was the dual use of delta-8-THC and delta-9-THC. One representative comment was: “It seems a lot more of a ‘functional’ high, at my job we call it work-weed. I get too much anxiety to effectively deal with customers on Delta 9, Delta 8 is just about perfect for when you gotta actually do things. I still do prefer Delta-9 after a long day though.” The eighth most common theme was adverse effects of delta-8-THC, for example: “I love Delta 8 because I do not need to take it daily. I’ve never had withdrawals when I did not take it. What I dislike about Delta 8 is the feeling of always being cold. I did read the dosage had something to do with this but unfortunately even reducing the dosage gave me the same result.” Participants also made comments that did not fit into the major themes. The most frequent of these comments was that delta-8-THC edibles or tinctures were more powerful than when delta-8-THC was inhaled as a vape: “How Delta 8 is consumed plays a large role in the effects, when eaten or taken in a tincture it feels much closer to Delta 9 in effects compared to when vaping/dabbing Delta 8.”

## Discussion

Participants’ reports were overall supportive of the use of delta-8-THC. Comparisons reveal that delta-8-THC experiences are primarily characterized by beneficial effects and are low in potentially adverse effects associated with cannabis use. Experiences of relaxation, pain relief, and euphoria were the most prominent, characterized as between “a moderate amount” and “a lot” on average. Participants reported “a little” of the cognitive distortions associated with delta-9-THC and cannabis use in general. Experiences such as an alerted sense of time, difficulties with short-term memory, and difficulty concentrating may not be problematic for consumers in certain contexts (e.g., relaxation and socialization), however they may in in others (e.g., operating a motor vehicle). Paranoia and anxiety are distressing mental states that may result from delta-9-THC ingestion (Freeman et al. 2015). On average, participants’ experiences of paranoia and anxiety were between “not at all” and “a little.” Experiences with delta-8-THC were characterized as less intense and with somewhat shorter duration than those with delta-9-THC.

Participant reports included a wealth of other information that can inform hypothesis testing and research questions in future studies. For example, it would be valuable to conduct systematic studies comparing experiences of delta-8-THC with delta-9-THC and pharmaceutical drugs. Participants viewed delta-8-THC experiences favorably in comparison, and most participants reported substitution of delta-8-THC for both delta-9-THC and pharmaceutical drugs, consistent with comparisons and substitutions of pharmaceuticals with cannabis products in general (Kruger and Kruger 2019; Lucas et al. 2016; Reiman et al. 2017). Participants reported being more active and productive with delta-8-THC than with delta-9-THC, and some suggested that delta-8-THC was more purely therapeutic than delta-9-THC. Participants also reported notable adverse experiences with delta-8-THC, most commonly that Delta-8-THC is harsher on the lungs than delta-9-THC when inhaled.

Some of the variation in experiences across individuals is likely due to inconsistencies in the products consumed, particularly in dosage, administration method, and impurities. Manufacturers have adjusted for the lower potency of delta-8-THC by increasing the dosage (e.g., 25 mg in edibles) relative to similar delta-9-THC products (where one dose has been defined as 10 mg) (Sideris et al. 2018; State of California Senate 2017). The US Cannabis Council tested 16 samples of non-cannabis-based products featuring delta-8-THC in April 2021 and found delta-9-THC levels ranging from 1.3 to 5.3% (well above the 0.3% level allowed in the 2018 Farm Bill), as well as heavy metals and unknown compounds in some of the samples (US Cannabis Council 2021). It is possible that substances other than Delta-8-THC contributed to both beneficial and adverse experiences in user reports.

### Policy considerations

The 2018 Farm Bill (U.S. Agriculture Improvement Act of 2018) created a legal loophole for the sale of hemp-derived delta-8-THC products in areas without legal adult use (i.e., recreational) and where the medical use of cannabis and cannabis products containing delta-9-THC requires medical authorization. Manufacturing and sales of delta-8-THC products skyrocketed due to greater accessibility to fulfill market demand. Yet, some states have made Delta-8-THC sales illegal. Paradoxically, of these 14 states, 6 states allow recreational delta-9-THC cannabis, 10 allow for medical delta-9-THC cannabis, and 3 have decriminalized recreational use of delta-9-THC cannabis.

The current study provides empirical evidence to inform discussions of delta-8-THC-related policies and practices. More research is needed to isolate the psychoactive effects of delta-8-THC and its possible therapeutic benefits, comparisons with pharmaceutical drugs and other cannabinoids, as well as risks and adverse effects. Such studies currently face considerable legal barriers, such as the Schedule I status of delta-9-THC. Banning delta-8-THC products while allowing the sale of delta-9-THC products seems inconsistent both in cannabis policy and in relation to our study results. Current results suggest that delta-8-THC products have therapeutic benefits and typical administration routes (consumption as an edible, tincture, or by vaping) may produce less harm than smoking cannabis flower. Vaping is considered a harm reduction solution (Fischer et al. 2017).

Harm reduction is a set of practical strategies and ideas aimed at reducing negative consequences associated with drug use (Marlatt et al. 2011). It is also a movement for social justice built on a belief in, and respect for, the rights of people who use drugs. Harm reduction has been widely used with various other substances, such as opioids (Rouhani et al. 2019), alcohol (Marlatt et al. 2011), and tobacco (Parascandola 2011). Interventions based on harm reduction principles have been successful in reducing risk behaviors related to cannabis use, for example driving while under the influence of cannabis (Poulin and Nicholson 2005). Although our results are largely descriptive, we provide an initial encouraging assessment of the suitability of the use of delta-8-THC as a possible harm reduction practice.

The U.S. Food and Drug Administration (FDA) has recommended collaborative research partnerships among academic researchers, government officials, and representatives from the cannabis industry to inform public health decisions related to cannabidiol (CBD), another cannabinoid with rapidly growing use (Hahn 2021). Similar research collaborations may accelerate the generation of knowledge regarding delta-8-THC and other cannabinoids.

## Limitations

This study compiled the self-reported experiences of delta-8-THC consumers. The patterns of experiences reported here require verification with carefully controlled studies, such as double-blind and randomized studies for comparisons of delta-8-THC with delta-9-THC and pharmaceutical drugs. The current study assessed participants’ naturalistic experiences, rather than experiences with a specific delta-8-THC product. Participants were recruited through the social networks of a delta-8-THC and CBD product manufacturer and a delta-8-THC social media interest group. Participant reports may be more enthusiastic than those of a randomly selected population-representative sample.

## Conclusions

Delta-8-THC products may provide much of the experiential and therapeutic benefits of delta-9-THC with lower risks and lesser adverse effects. Substitution of delta-8-THC for delta-9-THC may be consistent with harm reduction, one of the core principles of Public Health. The current study provided a broad descriptive assessment of self-reported experiences with delta-8-THC. Further systematic research will be critical in verifying the favorable reports of delta-8-THC consumers.

## Supplementary Information

**Additional file 1: Table S1.** Unique themes in responses to open-ended questions.

## Data Availability

The dataset used and analyzed for the current study are available from the corresponding author on reasonable request.

## Abbreviations

THC: Tetrahydrocannabinol
CBD: Cannabidiol
NASEM: National Academies of Sciences, Engineering, and Medicine
U.S.: United States
PTSD: Post-traumatic stress disorder
