# Supplementary material for: Delta-8-THC: Delta-9-THC’s nicer younger sibling?
Source: J Cannabis Res. 2022 Jan 4;4:4. doi: 10.1186/s42238-021-00115-8 (PMC8725316; doi:10.1186/s42238-021-00115-8)
Supplement: Supplementary file 1 — Additional file 1: Table S1. Unique themes in responses to open-ended questions. [file 42238_2021_115_MOESM1_ESM.docx]

**Table S1** Unique themes in responses to open-ended questions.

| **Theme/Subtheme** | |
| --- | --- |
| Comparisons between Delta-8 THC and Delta-9 THC | |
|  | Prefers flavor of Delta-8 THC |
|  | Prefers flavor of Delta-9 THC |
|  | Better content reliability with Delta-8 THC |
|  | Better content reliability with Delta-9 THC |
|  | Delta-8 THC stimulates the appetite more than Delta-9 THC |
|  | Delta-8 THC produces more anxiety than Delta-9 THC |
|  | Delta-8 THC effects lasted longer in initial use, then were similar in duration |
| Therapeutic effect or benefit from Delta-8 THC | |
|  | Fibromyalgia |
|  | Muscle relaxer |
|  | Nausea |
|  | Panic attack |
|  | Stress |
| Expressions of concern | |
|  | Concern over inconsistencies in product batches |
|  | Concern over potential market monopolization by large companies |
| Substitution of Delta-8 THC for other substances | |
|  | Benzodiazepines |
|  | Cannabidiol (CBD) |
|  | Melatonin |
|  | Non-steroidal anti-inflammatory drugs (NSAIDs) |
|  | Pharmaceuticals in general |
|  | Tobacco |
| Comparisons between Delta-8 THC and pharmaceutical drugs | |
|  | Delta-8 THC is better at pain relief |
|  | Delta-8 THC is less addictive |
|  | Delta-8 THC is better at treating anxiety |
|  | Delta-8 THC is better at treating headaches |
|  | Delta-8 THC is better at treating nausea |
|  | Delta-8 THC is better at treating arthritis |
|  | Delta-8 THC is better as a sleep aid |
|  | Delta-8 THC has fewer side effects |
|  | Delta-8 THC has less withdrawal |
| Dual use of Delta-8 THC and Delta-9 THC | |
|  | Delta-8 THC as a sleep aid and Delta-9 THC for recreation |
| Adverse effects of Delta-8 THC | |
|  | Nausea |
|  | Feeling cold |
| Other comments | |
|  | Desire for equivalency in Delta-8 THC and Delta-9 THC regulations |
|  | Unable to micro dose with Delta-8 THC |
|  | Individual differences in experiences with Delta-8 THC |
|  | Both medical and recreational use of Delta-8 THC |
|  | Delta-8 THC is better than CBD at treating pain. |

Note: See Table 2 for more common responses.
